# Mechanisms and Implications of Gut Microbiota-Derived Metabolites in the Regulation of Poultry Defensins

**DOI:** 10.3390/ani16152309

**Published:** 2026-07-26

**Authors:** Yifei Yu, Ke Xu, Yuqing Feng

**Affiliations:** State Key Laboratory of Animal Nutrition and Feeding, College of Animal Science and Technology, China Agricultural University, Beijing 100193, China

**Keywords:** poultry, defensins, microbial metabolites, regulatory mechanisms, immune modulation

## Abstract

The continuous intensification of modern poultry farming has confronted the industry with a dual crisis involving animal health and food safety. Host defensins, a major class of antimicrobial peptides (AMPs), exhibit broad-spectrum antimicrobial and immunomodulatory activities, demonstrating potential in disease prevention and immune support. By enhancing host innate defense mechanisms, they may complement existing antimicrobial strategies and contribute to reducing antibiotic dependence. Although accumulating evidence highlights the capacity of microbial metabolites to modulate defensin expression, a comprehensive and integrated understanding of the cross-talk between these microbial metabolites and host defensins remains lacking. To bridge this gap, this review summarizes the classification and biological functions of poultry defensins, while synthesizing recent advances in how microbiota-derived metabolites—such as short-chain fatty acids, amino acid derivatives, and secondary bile acids—regulate their expression. By elucidating the underlying receptor-mediated signaling pathways and mechanisms, this review provides strategic insights into leveraging microbial metabolites to stimulate endogenous poultry defensins and offers a reference for the future optimization of immunomodulatory strategies and the improvement of poultry health management.

## Simple Summary

With the rapid growth of the poultry industry and tightening global restrictions on antibiotic growth promoters, finding safe and effective alternatives has become an important research priority to sustain bird health and farm productivity. Antimicrobial peptides, particularly host-produced defensins, have attracted considerable interest due to their crucial roles in combating infections and modulating innate immunity. Emerging evidence indicates that symbiotic microbes in the poultry gut generate diverse metabolites that function as signaling molecules to modulate host defensin production. This review systematically categorizes poultry defensins and explores how gut microbiota-derived metabolites regulate their expression. By detailing these underlying molecular mechanisms, we hope to provide a theoretical framework for exploring metabolite-based nutritional strategies and to yield potential insights into enhancing endogenous host defenses, thereby advancing poultry production with less reliance on antibiotics.

## 1. Introduction

Amid rapid global population growth, the poultry industry has become a cornerstone of global food security by providing an efficient, high-yield source of animal protein. However, the rise of large-scale, intensive farming practices has introduced pressing environmental and food safety concerns, alongside an increased incidence of poultry diseases. Furthermore, high stocking density can impair immune function and exacerbate inflammatory responses, ultimately elevating disease susceptibility in poultry [1,2,3].

For decades, antibiotics have been widely used in poultry production for infection prevention and growth promotion [4]. However, the consequential surge in drug-resistant pathogens and antibiotic residues has precipitated a global public health crisis [5], threatening both human health and the sustainable development of the livestock industry. Driven by these concerns, numerous countries have enacted strict regulations banning antibiotic growth promoters in animal feed and restricting the therapeutic use of veterinary antibiotics. In 2006, the European Union implemented a comprehensive ban on antibiotic growth promoters in animal feed; on 9 July 2019, China’s Ministry of Agriculture and Rural Affairs issued Announcement No. 194, mandating the withdrawal of all growth-promoting medicated feed additives (with the exception of traditional Chinese medicine formulations) from the market by 1 January 2020 [6]. Consequently, AMPs have emerged as a promising alternative with substantial industrial potential, offering multi-faceted benefits that include promoting growth, improving intestinal health, bolstering immune development, and mitigating disease incidence [7].

Defensins represent evolutionarily conserved components of the innate immune system in multicellular organisms, widely distributed across animals, plants, and fungi. They exhibit diverse biological functions, including broad-spectrum antibacterial, antiviral, and antifungal activities, as well as potent immunomodulatory effects [8]. Studies have demonstrated that defensins protect host organisms from pathogen invasion by directly inactivating viruses [9], regulating inflammatory responses [10], and modulating cell cycles in tumor or pathogen infection models [11]. Therefore, the modulation of the expression of poultry defensins serves as a novel strategy to enhance disease resistance and provides a promising approach to boost innate immunity in poultry. However, it should be noted that overexpression of defensins may also exert detrimental effects on the host. For instance, elevated expression of human β-defensins has been associated with inflammatory diseases such as psoriasis, and under certain pathological conditions, their overexpression may even promote tumor signaling [12,13].

As a critical immune organ, the poultry gastrointestinal tract (GIT) serves as the central line of defense against pathogen invasion. During poultry growth and development, various microorganisms colonize the intestinal tract, establishing a complex microbiota that sustains a stable intestinal microecology. The commensal microbiota plays a pivotal role in maintaining intestinal barrier integrity and regulating immune homeostasis. Beyond direct cellular interactions with the microbiota, microbial metabolites act as major mediators modulating these physiological functions [14,15]. These metabolites exert profound effects on host immune metabolism through various mechanisms, including the activation of intracellular signaling pathways such as MAPK. In recent years, with advancing research into the cross-talk between the gut microbiota and host immunity, accumulating evidence supports that specific microbial metabolites can act as signaling molecules or ligands to precisely regulate the secretion and expression of host defensins. However, most current studies on the interplay between gut microbiota-derived metabolites and defensin regulation are derived from mammalian models, and direct evidence in poultry remains scarce. Moreover, even in mammals, it is often unclear whether the metabolites that induce defensin expression originate from microbial transformation or host metabolic pathways. Therefore, the regulatory mechanisms of gut microbiota-derived metabolites on defensins in poultry remain largely unexplored and urgently require systematic investigation.

## 2. Methods

This review delivers a comprehensive overview of the poultry gut microbiota, its diverse metabolites, and the underlying molecular mechanisms governing defensin regulation. Furthermore, we highlight preventive nutritional strategies based on microbial metabolite-mediated regulation of endogenous defensins. Ultimately, this review aims to provide new insights into enhancing disease resistance, developing innovative feed additives, and improving the quality and safety of poultry products.

This review was conducted as a narrative review of peer-reviewed literature examining the mechanisms and applications of microbial metabolites in the regulation of poultry defensins. A literature search was conducted in PubMed and Web of Science between February and July 2026, with particular emphasis on studies published over the past decade. Earlier seminal studies were also incorporated where contextually relevant. Search terms were organized into four thematic modules and their combinations: (i) Defensin/host defense peptides: “Defensin”, “Avian β-defensin”, “Host defense peptide”, “Antimicrobial peptide”; (ii) Gut microbiota and their metabolites: “Gut microbiota”, “Microbiome”, “Metabolite”, “*Salmonella*”, “short-chain fatty acids (SCFAs)”, “Butyrate”, “*Clostridium butyricum*”, “Tryptophan”, “Bile acid”, “Polyphenol”; (iii) Poultry-related: “Chicken”, “Poultry”, “Broiler”; and (iv) Plant extracts: “Quercetin”, “Curcumin”, “Resveratrol”. Both English and Chinese search terms were used to maximize retrieval efficiency on different platforms; however, only peer-reviewed articles published in English were included in the final review. Potentially relevant studies were identified through title and abstract screening, followed by full-text assessment for eligibility. Studies unrelated to defensins, host antimicrobial peptides, or intestinal biology were excluded. Evidence from in vivo poultry studies was prioritized throughout this review. However, where direct evidence in poultry was limited, mechanistic studies from non-poultry models (e.g., mouse, porcine, and human studies) were included as supportive evidence to provide biological and mechanistic context.

## 3. Classification and Functions of Poultry Defensins

### 3.1. Types of Poultry Defensins

**Figure 1:**
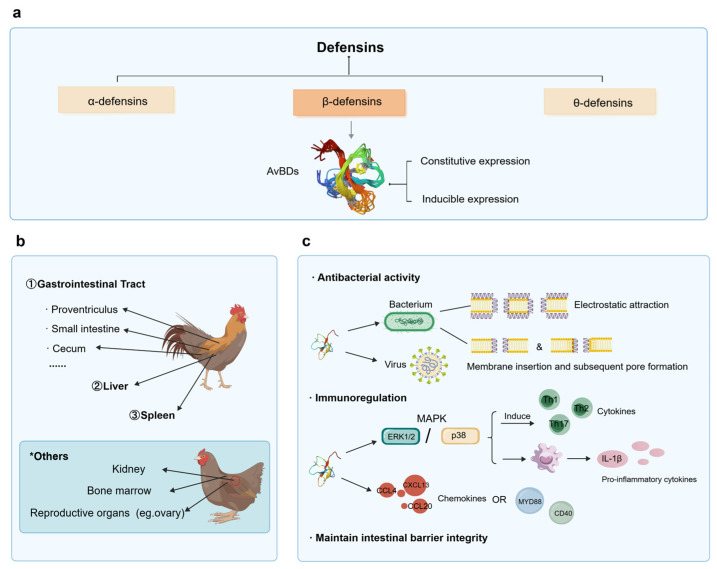
Classification and functions of defensins. (**a**) Detailed classifications of defensins. (**b**) Main distribution of defensins. * In addition to the gastrointestinal tract, the main distribution sites of AvBDs are found in other organs and tissues. (**c**) Biological functions of defensins. (Created with BioGDP.com [20]).

Based on the spatial arrangement of intramolecular disulfide bonds, vertebrate defensins can be classified into three major subclasses: α-, β-, and θ-defensins [16] (Figure 1a). Notably, poultry lack the typical α-defensins and θ-defensins found in other vertebrates, predominantly expressing β-defensins [17]. Emerging evidence indicates that avian β-defensins (AvBDs) can be categorized into three distinct groups based on their peptide homology and gene architecture: the first group features a short precursor peptide of 63–64 amino acid residues and an intron of less than 1.6 kb; the second group possesses a longer precursor sequence of approximately 68–69 residues and an intron exceeding 6.5 kb; and the final group consists of cysteine-stabilized cyclic peptides [18,19].

From a cellular origin perspective, poultry defensins are categorized into heterophil and non-heterophil peptides. Based on amino acid residue homology, heterophil defensins are further subdivided into two distinct subclasses: the first subclass shares 22 identical amino acid residues (e.g., THP-1 and CHP-1), whereas the second subclass shares 17 identical residues (e.g., THP-2 and Gal-2) [18,19].

The expression of AvBDs is generally classified into constitutive and inducible patterns, although this classification is not absolute (Figure 1a). Instead, their expression profiles are often influenced by experimental contexts such as specific tissue types, pathogens, and age. For example, previous studies have demonstrated that in uninfected chicken oviduct epithelial cells, *AvBD4*, *AvBD5*, *AvBD9*, *AvBD10*, *AvBD11*, and *AvBD12* are persistently expressed at high levels, representing typical constitutive expression, while *AvBD1*, *AvBD3*, *AvBD13*, and *AvBD14* exhibit moderate expression. In contrast, *AvBD2*, *AvBD6*, *AvBD7*, and AvBD8 are expressed at low levels and can be markedly induced upon *Salmonella* Enteritidis (SE) infection, showing characteristic inducible features [21]. Nevertheless, AvBD expression patterns are not static and exhibit complex pathogen-specific responses. A specific AvBD that is strongly induced by one pathogen may remain unaltered or even suppressed upon infection with another pathogen. For instance, in broilers infected with *Salmonella* Pullorum in the gastrointestinal tract, the expression of *AvBD3*, *AvBD4*, *AvBD5*, *AvBD6*, and *AvBD12* is significantly upregulated, indicating an inducible type, whereas *AvBD1*, *AvBD2*, *AvBD7*, *AvBD8*, and *AvBD9* show no obvious changes, and *AvBD10*, *AvBD11*, *AvBD13*, and *AvBD14* are even significantly downregulated, suggesting a constitutive type [22]. Host age is another critical factor shaping AvBD expression patterns. It has been reported that in sexually mature 52-week-old chicken ovaries, the expression of *AvBD4*, *AvBD5*, *AvBD7*, and *AvBD11* is significantly higher than that in immature 12-week-old chickens, and these genes can be further strongly induced following SE infection. In contrast, SE infection exerts no significant effect on the expression of these genes in 104-week-old chicken ovaries [23]. Furthermore, AvBD expression varies substantially across tissues, exhibiting distinct tissue specificity. For example, *AvBD11*, which is abundantly expressed in the isthmus epithelial cells of the hen oviduct, is barely detectable in the gastrointestinal tract [21,24]. Overall, these findings demonstrate that AvBD expression patterns are highly dynamic and context-dependent, and their biological functions should be interpreted strictly based on specific experimental conditions.

As the most extensively characterized poultry species to date, chickens possess 14 identified AvBDs, designated as AvBD1 through AvBD14 [24,25]. These AvBDs are widely distributed across diverse tissues, including the bone marrow, bursa of Fabricius, spleen, respiratory tract, kidneys, and reproductive organs (Figure 1b). Notably, while the majority of AvBDs play pivotal roles within the digestive tract, *AvBD11* is unique due to its minimal to undetectable expression in the GIT, liver, and spleen [24].

### 3.2. Biological Functions of Poultry Defensins

#### 3.2.1. Antimicrobial Activity

A hallmark feature of AvBDs is their broad-spectrum antimicrobial activity, exerting potent bactericidal effects against Gram-positive bacteria, Gram-negative bacteria, and fungi (Figure 1c). Taking AvBD9 as an example, studies have confirmed that concentrated supernatant containing AvBD9 exhibits antimicrobial activity against a range of bacteria, including *Escherichia coli*, *Salmonella* Paratyphi, *Salmonella* Pullorum, *Pseudomonas aeruginosa*, *Enterococcus faecalis*, and *Enterobacter cloacae*. In broiler feeding trials, AvBD9 treatment significantly increased average daily gain and immune organ indices [26]. Mechanistically, AvBDs mirror conventional AMPs by primarily targeting and disrupting the bacterial cell membrane; they eradicate pathogens through a multi-step membrane permeabilization process driven by electrostatic attraction, membrane insertion, and subsequent pore formation [27].

Furthermore, recent studies have confirmed that certain AvBDs possess antiviral activity. For example, duck AvBD3C exhibits antiviral activity against the H5N1 subtype of avian influenza virus (AIV), while chicken AvBD2 inhibits the H1N1 subtype. However, this antiviral activity is not universal among all AvBDs. Chicken AvBD7 demonstrates no significant inhibitory effect against the H1N1 subtype of AIV, though its efficacy against other viral strains remains to be elucidated. Currently, most evidence for the antiviral activity of AvBDs comes from in vitro studies, with relatively limited direct in vivo evidence available; therefore, their physiological relevance remains unclear. Moreover, existing research has largely focused on only a few defensins in chickens, and the evidence for avian defensins as antiviral agents is not yet universal. Beyond direct antiviral action, AvBDs can act indirectly; chicken AvBD11 induces cytokine expression in chicken erythrocytes, functioning as an immunomodulator to confer resistance against the H9N2 subtype of AIV [28].

#### 3.2.2. Immunomodulatory Activity

Beyond direct pathogen elimination, poultry defensins amplify pro-inflammatory responses and accelerate pathogen clearance by activating specific intracellular cascades and inducing cytokine expression (Figure 1c). In chicken macrophage cell lines, AvBD8 activates the mitogen-activated protein kinase (MAPK) pathway via ERK1/2 and p38 signaling molecules, thereby stimulating the secretion of pro-inflammatory cytokines such as IL-1β [29]. Similarly, chicken AvBD5 triggers the activation of various cytokines associated with Th1, Th2, and Th17 immune responses in macrophages, a process also regulated through ERK1/2- and p38-mediated MAPK signaling [30]. Additionally, AvBDs regulate an array of downstream immune-related molecules. Chicken AvBD8 induces the expression of chemokines like CCL4, CXCL13, and CCL20 [29], while AvBD5 upregulates MyD88 and CD40 [30]. In the disease model of necrotic enteritis, defensins participate directly in IL-17-mediated anti-infective immunity. Notably, the overexpression of IL-17 significantly upregulates the expression of *AvBD1*, *AvBD2*, *AvBD4* and *AvBD6* in the small intestine of broilers [31], ultimately mitigating intestinal inflammation. Together, these findings highlight that poultry defensins and cytokines intersect to form a coordinated, multi-level immunoregulatory network.

#### 3.2.3. Maintenance of Intestinal Barrier Integrity

As an integral component of the mucosal protective barrier, AvBDs play crucial physiological roles in maintaining the structural integrity and homeostasis of the gut (Figure 1c). In a *Clostridium perfringens* infection model, AvBDs are widely upregulated throughout the intestine to orchestrate host defense [31]. Similarly, in a low-dose *Campylobacter jejuni* infection model, intestinal epithelial cells increase the transcript levels of AvBDs such as *AvBD1*, *AvBD6*, and *AvBD10* to resist bacterial colonization [32]. Furthermore, in an *Escherichia coli* infection model, chicks pretreated with baicalin displayed significantly elevated mRNA levels of *AvBD1*, *AvBD2* and *AvBD4*. This upregulation effectively lowered the diarrhea rate, alleviated pathological lesions in intestinal tissues following avian pathogenic *E. coli* challenge, and fortified overall intestinal barrier function [33]. Beyond the gut, AvBDs contribute similarly to maintaining the homeostasis of other mucosal tissues, such as the reproductive tract.

## 4. Microbial Metabolites in Poultry Gastrointestinal Tract

The gut microbiota and their derived metabolites collectively regulate intestinal morphogenesis and physiological functions [34,35]. Consequently, shifts in microbial community structure are invariably accompanied by alterations in their metabolic profiles. As major mediators of host–microbe interactions [36], the types and functions of these metabolites are linked to specific microbial taxa.

The gut microbiota composition of healthy poultry is generally complex and varies significantly across different intestinal segments. Burrows et al. revealed that at the genus level, *Lactobacillus* is the most prominent genus across all intestinal segments except the cecum. In the ileum, *Lactobacillus*, *Candidatus arthromitus*, and *Faecalibacterium* are present at relatively high abundance. The jejunum is dominated by *Faecalibacterium* and *Stenotrophomonas*, whereas the cecum is mainly enriched with *Faecalibacterium*, *Eisenbergiella*, and *Oscillibacter*. Overall, *Lactobacillus* and Faecalibacterium are considered core microbial members commonly detected in the poultry GIT [37].

At the phylum level, the poultry GIT is predominantly colonized by Firmicutes, followed by Proteobacteria and Bacteroidetes. In addition, Actinobacteria (*Bifidobacterium*) and Proteobacteria (*Desulfohalobium)* maintain detectable abundance in the gut microbiome [37]. Other rare phyla, including Verrucomicrobia (e.g., *Akkermansia*) [38], also contribute to the overall composition of poultry GIT microbiota.

Furthermore, Burrows et al. demonstrated that the relative proportion of *Lactobacillus* gradually decreases with increasing poultry age [37]. Pin Viso et al. found that dietary tannin supplementation leads to an increased Firmicutes/Bacteroidetes ratio [39]. Multiple studies have confirmed that a variety of factors, including poultry breed, age, geographic location, rearing system, and feeding regimen, can substantially modulate the composition of poultry GIT microbiota.

Through diverse metabolic activities, the indigenous microbiota within the poultry GIT produces a series of critical bioactive molecules, including lactate, SCFAs, amino acids, and their derivatives, alongside other microbial products (Table 1).

### 4.1. Lactic Acid

Lactic acid is a key microbial metabolite of the poultry gut, predominantly synthesized by Firmicutes taxa such as *Lactobacillus*. Actinobacteria also contribute significantly to lactate production. For instance, *Bifidobacterium* breaks down carbohydrates through heterolactic fermentation to yield acetate and lactate [55], which effectively inhibits pathogen proliferation and enhances intestinal barrier function.

### 4.2. Short Chain Fatty Acids

As the primary class of microbial metabolites [60], SCFAs alleviate intestinal inflammation through multiple mechanisms, including activating G-protein coupled receptors (GPRs), inhibiting histone deacetylases (HDACs), and downregulating pro-inflammatory cytokines [61,62]. SCFAs are mainly produced by taxa belonging to the phyla Bacteroidetes, Firmicutes, and Actinobacteria. Notably, butyrate—a major SCFA—can induce the expression of multiple AvBDs, thereby mitigating intestinal inflammation and modulating immune functions.

### 4.3. Amino Acid Metabolites

As essential energy sources and metabolic precursors for the gut microbiota, amino acids play an indispensable role in animal growth, development, and the maintenance of homeostasis. Accumulating evidence suggests that microbiota-derived amino acids constitute a major proportion of the total amino acid pool within the GIT; notably, over 60% of proteins in the distal ileum of poultry originate from microbial biomass.

Tryptophan and other amino acid derivatives represent key intestinal microbial metabolites primarily generated by Bacteroidetes and Firmicutes [63,64]. Downstream derivatives such as tryptamine and indole play crucial roles in maintaining intestinal homeostasis and regulating host defensin expression [15,65,66].

### 4.4. Vitamins and Cofactors

Vitamins and cofactors are among the principal metabolites synthesized by Actinobacteria and Firmicutes [15,55]. Crucially, Proteobacteria—a phylum harboring various pathogens such as *Salmonella* and *Escherichia*—is typically restricted to a tightly controlled low abundance in the healthy GIT. However, under stress conditions or when GIT homeostasis is disrupted, Proteobacteria can proliferate opportunistically, leading to severe inflammation.

## 5. Regulation of Defensin Expression by Microbial Metabolites and Underlying Mechanisms

### 5.1. Regulation of Defensins by Amino Acids

Recent advances have increasingly confirmed that amino acids and their derivatives function as key signaling molecules to modulate defensin expression in animals. Although most mechanistic data are obtained from mammalian models, resulting in a shortage of direct experimental evidence in poultry, these research findings still offer important references for studies on poultry defensins, and the relevant regulatory mechanisms are likely conserved among different species.

#### 5.1.1. Tryptophan and Its Metabolites

Tryptophan is an essential amino acid that undergoes extensive catabolism by the gut microbes within the GIT [67]. The microbial breakdown yields diverse bioactive derivatives, including indole and its derivatives [68], and kynurenine [69], which collectively orchestrate host mucosal immunity and disease resistance by modulating defensin production.

A primary mechanism through which tryptophan metabolites regulate host defensins is the activation of the aryl hydrocarbon receptor (AhR). For instance, kynurenine has been shown to upregulate β-defensin-2 (BD-2) expression in the murine jejunum and ileum via AhR binding [69]. Similarly, in mouse models, indole-3-carbinol functions as an AhR ligand to induce BD-1 expression in colonic epithelial cells, an effect completely abolished upon AhR gene knockout [70]. Beyond the AhR pathway, tryptophan derivatives utilize alternative cascades to modulate defensins. In porcine models, tryptophan and kynurenine can activate the calcium-sensing receptor (CaSR)-AMPK pathway in a CaSR-dependent manner, thereby elevating pBD1 and pBD2 levels and mitigating LPS-induced intestinal inflammation [71]. Furthermore, in rat models, tryptophan stimulates BD-2 synthesis in the rat intestinal mucosa by triggering the intracellular mammalian target of rapamycin (mTOR) pathway [69].

Overall, direct evidence regarding the regulation of poultry defensin expression by tryptophan and its metabolites remains extremely limited. Existing studies in poultry have primarily focused on the beneficial effects of tryptophan on immune organ development and serum immunoglobulin levels [72], without directly targeting defensins as a research objective. Evidence from mammalian studies may provide a hypothetical basis for avian research to some extent; however, the conservation of these mechanisms in poultry remains to be experimentally validated.

#### 5.1.2. Arginine

In addition to tryptophan, other amino acids also participate in the orchestration of defensin expression. L-arginine plays a critical role in host–microbe interactions. As a major metabolite in both mammals and gut microbes, it also serves as a dietary supplement, participating in multiple metabolic pathways and modulating immune function [73]. Studies in porcine models have revealed that L-arginine suppresses the TLR4/NF-κB and MAPK pathways, potentially upregulating the expression of porcine intestinal β-defensins to alleviate inflammation and enhance disease resistance. Moreover, a novel regulatory axis has been proposed wherein L-arginine stimulates porcine epithelial β-defensin expression through the activation of the downstream mTOR pathway [74]. Currently, the evidence for the above mechanisms is mainly derived from mammalian models, and whether these mechanisms are conserved in poultry remains to be verified. However, the L-arginine used in these studies was derived from exogenous supplementation. To date, research on microbiota-derived arginine in regulating the animal gut, particularly in poultry, remains largely unexplored and warrants further experimental investigation.

#### 5.1.3. Branched-Chain Amino Acids

Branched-chain amino acids (BCAAs) represent another class of essential amino acids critical for host development and immunity. Studies have shown that BCAAs exist in the animal intestine both as microbial metabolites and as important dietary sources. Within the poultry ileum, microbiota-derived BCAAs—particularly leucine—occupy a prominent portion of the luminal pool, mirroring their significant contribution to meeting the BCAA requirements. However, the precise mechanisms by which microbiota-derived BCAAs regulate host defenses remain enigmatic and warrant deeper investigation. To date, preliminary studies indicate that in porcine models, dietary supplementation with BCAAs elevates the relative mRNA expression of β-defensins in the small intestine and ileum of weaned piglets. Specifically, leucine, isoleucine, and valine promote β-defensin synthesis by activating the Sirt1/ERK/90RSK pathway [75]. Concurrently, in human colonic cells, exogenous supplementation with isoleucine has been shown to induce BD-2 expression by activating the G protein-coupled receptor-ERK cascade in intestinal epithelial cells [76]. The aforementioned mechanistic evidence is mainly derived from porcine and human cell models. The direct effects of BCAAs on the regulation of poultry defensins, as well as the contribution of microbiota-derived BCAAs, still lack experimental validation and warrant further investigation. These dietary BCAA-mediated regulatory mechanisms offer a valuable theoretical reference for future exploration into how microbiota-derived BCAAs modulate avian immunity.

### 5.2. Regulation of Animal Defensins by SCFAs

SCFAs—primarily acetate, propionate, and butyrate—are the principal end products of dietary fiber or complex carbohydrate fermentation by the gut microbiota [77]. Acetate is predominantly synthesized by members of the phyla Firmicutes [78], Actinobacteria [55], and Bacteroidetes [79]. Propionate production is largely attributed to specific genera within Firmicutes (e.g., *Megamonas*, *Phascolarctobacterium* and *Blautia*) as well as the majority of Bacteroidetes [80,81]. Meanwhile, butyrate is primarily generated by Firmicutes such as *Clostridium butyricum*, *Faecalibacterium* and *Eubacterium* [82].

Among the major SCFAs, butyrate exerts the most profound regulatory impact on host defensins. In poultry production, butyrate is widely utilized as a feed additive to enhance innate immunity and pathogen resistance. In vitro experiments by Sunkara et al. confirmed that in models such as chicken macrophages (HD11), butyrate induces the expression of various chicken defensins, thereby enhancing the antimicrobial activity of monocytes against *Salmonella* Enteritidis. Furthermore, in vivo experiments from the same study further demonstrated that butyrate significantly induces *AvBD9* expression in the chicken crop [83]. In addition, a series of in vitro (chicken HD11 cells) and in vivo experiments have shown that butyrate exhibits strong synergistic interactions with other compounds. For instance, Robinson et al. demonstrated that butyrate and forskolin synergistically upregulate *AvBD3*, *AvBD8*, *AvBD9* and *AvBD10* expression in a concentration- and time-dependent manner, yielding an induction far exceeding the cumulative effects of either agent alone; the study also found that this combination upregulates *AvBD2*, *AvBD6* and *AvBD7* [84]. Similarly, studies by Sunkara et al. confirmed that combinations of butyrate with forskolin or cyclic AMP synergistically augment *AvBD9* expression [85]. Mechanistically, inhibition of the MEK-ERK MAPK pathway enhances this synergistic induction, whereas blockade of the JNK or p38 MAPK cascades drastically dampens *AvBD9* expression induced by butyrate or forskolin alone [85]. Dietary sugars also display synergy with butyrate; in models such as chicken macrophages (HD11), lactose and butyrate co-regulate *AvBD9* transcription via the MAPK, NF-κB, and cAMP pathways, while exerting negligible effects on *AvBD14* [86]. Additionally, co-stimulation with these compounds prompts more pronounced histone hyperacetylation than single treatments, though the exact epigenetic mechanisms require further elucidation [86].

The regulation of defensins by SCFAs also exhibits a chain-length effect. Studies reveal that butyrate serves as a highly potent inducer of AvBDs in chicken HD11 macrophages and primary monocytes, with the induction intensity of AvBDs being generally negatively correlated with the hydrocarbon chain length of free fatty acids [87]. However, this chain-length effect varies across different cell types. For example, studies have shown that long-chain fatty acids exhibit weaker induction in HD11 macrophages, but still retain some induction capacity in primary monocytes. The same study also found that the presence of double bonds in the hydrocarbon chain appears to enhance the ability of fatty acids to induce HDP expression, suggesting that the saturation status of the hydrocarbon chain may also influence the chain-length effect. Among SCFAs, although acetate and propionate also possess biological functions, butyrate is widely recognized as the most prominent molecule for inducing avian defensin activity, and the three SCFAs exhibit marked differences in their effects on host defense peptide regulation. In terms of individual SCFAs, butyrate demonstrates the strongest ability to induce *AvBD9* gene expression, followed by sodium propionate and acetate. Although acetate and propionate alone have limited defensin-inducing capacity, their combination with butyrate exerts a significant synergistic effect. In poultry in vivo feeding models, compared with single dietary SCFA, the combined application of acetate, propionate and butyrate exhibits strong synergistic effects, which further enhances *AvBD9* induction and reduces *Salmonella* colonization in the chicken cecum [87].

In in vitro murine intestinal epithelial models, SCFAs have been shown to mediate β-defensin expression by activating mTOR and STAT3 pathways via GPR43 [88]. In the porcine 3D4/2 macrophage in vitro model, butyrate can also upregulate endogenous HDPs through HDAC inhibition activity [89]. In contrast, propionate and butyrate may exert inhibitory effects on defensins in a dose-dependent manner. In a human colonic epithelial cell model, propionate and butyrate suppressed the transcription of α-defensin 5 at the highest concentration of 9 mM. The same study also demonstrated a strong inhibitory effect of lactate on α-defensin 5 gene expression [90]. Notably, the above regulatory evidence is exclusively derived from mammalian in vitro models and has not yet been validated in poultry. The signaling mechanisms identified in mammalian models may provide novel insights for further research in poultry.

### 5.3. Regulation of Animal Defensins by Secondary Bile Acids

Bile acids are steroid molecules synthesized from cholesterol in the liver. Upon entering the intestine via the bile duct, primary bile acids are biotransformed by the gut microbiota into secondary bile acids (SBAs). Functioning as potent signaling molecules, SBAs modulate immune responses along the gut-liver axis [8]. In poultry, SBAs exist in both free and conjugated forms. Free forms include deoxycholic acid (DCA) and lithocholic acid, whereas conjugated forms comprise taurodeoxycholic acid and taurolithocholic acid, with taurine-conjugated species being predominant in poultry [8,91].

Kim et al. demonstrated that in chicken jejunal explants and HD11 macrophages, DCA and sodium butyrate (NaB) synergistically induce the expression of *AvBD3* and *AvBD9*. However, this synergistic induction was not universal, as *AvBD6*, *AvBD7*, and *AvBD10* were not co-induced [92].

While the exact regulatory pathways of SBAs in poultry currently remain to be fully characterized, parallel mechanisms in mammals are well documented. In vitro experiments demonstrate that DCA stimulates the release of human BD-1 (HBD-1) and human BD-2 (HBD-2) in epithelial monolayers and colonic mucosal tissues via the Takeda GPCR5 (TGR5)/NF-κB pathway [93]. In cholestatic mouse models, bile acids and bilirubin mediate HBD-1 expression by activating the farnesoid X receptor (FXR) and the constitutive androstane receptor [94]. Relevant findings obtained from mammalian studies may provide testable hypotheses regarding the potential mechanisms by which SBAs regulate AvBDs in poultry. Nevertheless, whether these pathways are conserved in poultry still awaits experimental verification.

### 5.4. Regulation of Defensins by Microbial Metabolites Derived from Plant Extracts

**Figure 2:**
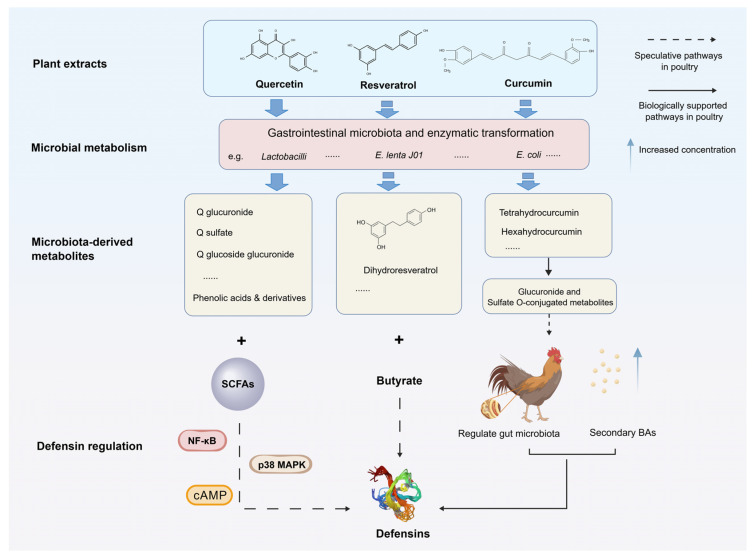
Regulation of defensins mediated by microbiota conversion of plant extracts (Created with BioGDP.com [20]).

Owing to their natural, safe, and multifunctional properties, plant extracts are widely used as feed additives to improve poultry health and growth performance. However, many active phytochemical components exhibit low direct bioavailability in the avian gut, requiring biotransformation by the gut microbiota to unlock their full biological activities and effectively modulate host immunity and defensin expression (Figure 2) [95].

The bioavailability of plant polyphenols is regulated by multiple factors, including digestion, absorption, and metabolism [96]. Most natural polyphenols exist in the diet primarily as esters, glycosides, or polymers, and are rarely utilized directly by animals; they must undergo metabolic conversion through the action of gastrointestinal microbiota and enzymes before absorption [97]. It is estimated that only approximately 5–10% of structurally simple free polyphenols and some glycosides can be directly absorbed via hydrolysis in the small intestinal epithelium [98], whereas the vast majority of unmodified and conjugated polyphenols rely on gut microbial catabolism [99]. In poultry, polyphenols display remarkably low bioavailability in the GIT. Approximately 90% of ingested phenolic compounds escape proximal absorption and enter the distal gut, where the resident microbiota enzymatically cleaves their core structures into low-molecular-weight metabolites with enhanced bioavailability and distinct bioactivities [100,101].

Quercetin serves as a prime example; it is poorly absorbed directly by the poultry intestine and is instead degraded into glucuronic acid and various phenolic acids by gut microbiota such as *Lactobacillus* to exert its biological functions [100,102]. Dietary supplementation with quercetin or quercetin nanoparticles markedly upregulates *AvBD6* and *AvBD12* mRNA expression in the intestinal tissues of broilers challenged with *C. perfringens* [103]. Quercetin nanoparticles also increase intestinal *AvBD6* and *AvBD12* expression in laying hens in a dose-dependent manner [104]. Furthermore, in vitro investigations using chicken HTC macrophages have demonstrated that quercetin can synergistically induce *AvBD9* secretion when combined with SCFAs via the NF-κB/p38 MAPK/cAMP axis [105]. However, the above studies only reported the correlation between quercetin supplementation and the upregulation of AvBDs, and did not directly explore whether microbial metabolites of quercetin mediate this effect. The regulatory role of microbiota-derived quercetin metabolites in AvBD expression remains to be elucidated. Hence, flavonoid extracts like quercetin likely undergo microbial transformation into glycosides or phenolic acids prior to modulating AvBDs.

Similarly, other polyphenols such as resveratrol and anacardic acid enhance avian defensin expression and exhibit synergy with butyrate. Resveratrol possesses extremely low bioavailability and relies on transformation by specific intestinal strains, such as *Eggerthella lenta* J01, into active metabolites like dihydroresveratrol [106]. However, evidence supporting this transformation is mainly derived from mouse models and in vitro experiments, and the conservation of such metabolic pathways in poultry remains to be verified. Similar to quercetin, resveratrol upregulates *AvBD9* expression in a dose-dependent manner in in vitro studies using chicken HTC macrophages, and exhibits significant synergistic effects with butyrate on *AvBD9* gene expression. These findings highlight the potential of combining natural phenolic COX-2 inhibitors with butyrate as novel immunotherapeutic strategies [105]. Nevertheless, it still remains unknown whether the induction of defensins by resveratrol is mediated by its microbial metabolites. The microbial degradation pathways of anacardic acid also remain largely unknown and require further investigation.

Additionally, plant extracts can indirectly induce AvBD production by modulating the gut environment. For example, curcumin modulates chicken gut microbiota composition and alters the abundance of SBA, thereby regulating defensin expression and alleviating intestinal inflammation [107]. Curcumin can also be converted by gut microbes such as *E. coli* into various active metabolites, including tetrahydrocurcumin, through the generation of corresponding glucuronide- and sulfate-conjugated metabolites [108] to exert its biological activity. Notably, this transformation has only been validated in mammalian models, and whether it occurs similarly in poultry remains to be explored.

In addition to the low bioavailability of plant extracts in poultry diets, differences in bioactive compound concentrations and the gap between in vitro experimental effects and actual in vivo outcomes also limit research progress on plant extracts in the poultry field. Specifically, the stability and bioactive components of plant extracts are influenced by conditions such as plant variety, growing region, harvest time, extraction process, and storage, making standardized research difficult [109]. These factors may collectively result in the failure to replicate in vitro findings on the regulation of animal defensins by plant extracts within the complex digestive environment of poultry in vivo. Therefore, research targeting avian species remains considerably challenging.

### 5.5. Regulation of Defensins by Other Metabolites

In addition to the aforementioned major metabolite classes, the gut microbiota synthesizes an array of other bioactive molecules that directly or indirectly influence defensin expression. Structural components such as peptidoglycan have been shown to markedly induce poultry defensins. The peptidoglycan of *Lactobacillus rhamnosus* can activate NF-κB or p38 MAPK and JNK pathways through TLR2 or NOD1 receptor mediation, thereby upregulating *AvBD9* expression without provoking a detrimental inflammatory response [110].

Distinct from the molecules described above, 5-hydroxytryptamine (5-HT) generally exerts an inhibitory effect on defensin expression. Research demonstrates that 5-HT treatment downregulates mBD-1 and mBD-3 expression. Mechanistically, 5-HT binds to the 5-HT7 receptor and suppresses peroxisome proliferator-activated receptor γ, a transcription factor essential for maintaining colonic β-defensin expression, and further diminishes defensin secretion through an ERK1/2-dependent pathway [111].

In summary, amino acids, SCFAs, SBAs, plant extract-derived microbial metabolites, and various structural components regulate animal defensins through distinct, multifaceted mechanisms. Tryptophan promotes the expression of defensins like BD-2 and pBD1 by generating kynurenine and indole to activate the AhR, CaSR-AMPK, and mTOR signaling pathways. Arginine enhances porcine β-defensin secretion by activating the mTOR pathway or suppressing the TLR4/NF-κB and MAPK cascades. BCAAs upregulate porcine β-defensins via the Sirt1/ERK/90RSK pathway and enhance human BD-2 expression via the G protein-coupled receptor-ERK pathway. SCFAs, led by butyrate, modulate multiple AvBDs through unique carbon chain-length effects and synergistic cross-talk with forskolin, cAMP, and DCA. SCFAs also induce β-defensin expression by activating mTOR and STAT3 pathways via GPR43 receptors, while HDAC inhibition represents another key mechanism for butyrate-mediated HDP regulation in swine. SBAs stimulate human β-defensin secretion via the TGR5/NF-κB axis and mediate HBD-1 expression via FXR and CAR receptors in mice. Plant extracts exert direct or indirect inductive effects on AvBDs following microbial degradation and modification of the microbial ecosystem. Finally, other bacterial structural components and host molecules facilitate defensin secretion by boosting cascades like NF-κB, whereas 5-HT suppresses defensin expression through the ERK1/2 pathway (Table 2).

However, the above mechanisms also highlight the current limitations of the available evidence. The vast majority of mechanistic studies are derived from mammalian models, and the validation of complete signaling pathways in poultry remains markedly insufficient. Moreover, existing research has largely focused on the independent effects of single metabolites, while systematic analyses of the synergistic effects between different metabolic pathways, dose–response relationships, and species-specific regulatory patterns are lacking. Therefore, although current studies have preliminarily established the plausibility of microbial metabolite-mediated defensin regulation, their applicability in poultry and the precise regulatory mechanisms involved still require further validation and integration.

## 6. Applications and Outlooks

As core elements of the host innate immune system, animal defensins serve as essential barriers against pathogen invasion and are vital for maintaining intestinal microecological stability. Harnessing host- or microbiota-derived metabolites to regulate these peptides presents a promising nutritional or immunomodulatory strategy.

In modern biotechnology, the direct production of exogenous defensins via recombinant fermentation for feed supplementation often faces commercial bottlenecks, including high industrial manufacturing costs and high susceptibility to proteolytic degradation within the host digestive tract. In contrast, stimulating endogenous defensin expression by modulating the gut microbiota and their metabolic profiles, or via the direct dietary administration of these metabolites, offers superior stability and feasibility. However, this metabolite-mediated regulatory strategy still faces several challenges in practical application.

First, the stability of metabolites warrants further investigation. For instance, microbial metabolites may be rapidly absorbed in the proximal intestine, making it difficult to achieve effective concentrations at target sites; plant-derived bioactive components generally have low bioavailability and often require formulation improvements to enhance their efficacy. Second, the overexpression or uncontrolled induction of defensins may inadvertently impair animal health and increase production costs. In poultry, investigations have revealed that the upregulation of *AvBD10* is accompanied by a significant reduction in body weight [112]. Furthermore, lentiviral vector-mediated transduction of the HNP4 gene has been reported to compromise hatchability in chickens [113], directly impacting hatchery efficiency. Therefore, precisely identifying and establishing the safe physiological thresholds for defensin expression is paramount for their future practical application. In addition, host genetic background, age, individual differences in gut microbiota, and environmental factors may all influence the regulatory effects of metabolites, making it difficult to achieve universal application strategies. The higher safety requirements for metabolites as immunomodulators, along with new regulatory and approval frameworks, also represent specific issues that need to be addressed for the future development of the poultry industry.

Although numerous studies have uncovered the regulatory potential of microbial metabolites on host defensins, the specific mechanisms governing defensin regulation in poultry remain largely understudied compared with mammalian models. The interactive cross-talk between poultry defensins and metabolites like tryptophan or BCAAs requires further exploration. As poultry-specific regulatory networks are progressively uncovered and validated, AvBD-targeted strategies are expected to provide new insights for precision nutrition and disease prevention. In practical poultry production, these strategies may help reduce the incidence of intestinal diseases such as necrotic enteritis, enhance immune function, improve growth performance and feed efficiency with a lower feed conversion ratio, and optimize reproductive performance in breeder flocks, thereby improving poultry product quality. Ultimately, this approach will provide critical theoretical and technical support for sustainable poultry production, successfully aligning animal health with environmental and economic benefits.

## 7. Conclusions

In conclusion, gut microbiota-derived metabolites serve as vital bridges in host–microbe interactions and play an indispensable role in regulating the expression of poultry defensins. This review systematically summarized how diverse categories of microbial metabolites—including SCFAs, tryptophan and other amino acid derivatives, SBAs, and transformed plant extracts—modulate AvBDs. These metabolites operate through intricate, receptor-mediated signaling networks (such as AhR, mTOR, MAPK, and NF-κB pathways) or epigenetic modifications like HDAC inhibition. Harnessing these microbial metabolites to stimulate endogenous poultry defensins may offer a promising and sustainable novel strategy for future disease prevention and immune enhancement. However, their economic feasibility and broad applicability in the poultry industry still require further validation. Additionally, although metabolite-mediated endogenous defensin regulation strategies hold potential application value, defensin overexpression may also incur significant physiological safety costs. Therefore, before these metabolite-driven immunomodulatory strategies can be commercialized, identifying and maintaining a safe physiological threshold for defensin expression, as well as balancing immune enhancement with host physiological homeostasis, are essential prerequisites. Future research urgently needs to systematically explore the safe range of defensin expression to provide a theoretical foundation for developing antibiotic-free nutritional strategies that achieve both efficacy and animal health.

On the other hand, research on avian defensins is subject to notable limitations, with several critical research gaps awaiting further investigation. First, a large portion of the current mechanistic understanding is derived from mammalian models; thus, more poultry-specific in vivo studies are needed to elucidate direct avian signaling pathways more thoroughly and reveal the precise interplay between specific AvBDs and individual metabolites. Second, the potential trade-offs between hyper-activated innate immunity (e.g., defensin over-expression) and growth performance or hatchability demand careful optimization to establish safe physiological thresholds. Future efforts should leverage advanced multi-omics approaches—combining metagenomics, metabolomics, and transcriptomics—to map out the precise host-metabolite-defensin network. This may provide a theoretical basis for exploring metabolite-related nutritional regulation and host defense modulation, which could potentially assist in improving poultry health and reducing reliance on antibiotics in modern poultry farming. By systematically constructing the regulatory network between metabolites and defensins, future research may gain new insights into identifying key immunomodulatory metabolites and developing targeted nutritional interventions. Such approaches could upregulate beneficial defensin expression while avoiding the risk of excessive inflammation, thereby achieving an optimal balance between poultry health maintenance and production performance. Ultimately, this research may provide a novel solution pathway based on microbiota and metabolite interactions for reducing antibiotic use, while also contributing practically to lowering disease incidence, improving feed conversion efficiency, and enhancing reproductive performance in poultry production.

## Figures and Tables

**Table 1:** Gut microbiota-derived metabolites and their immunoregulatory functions in poultry *.

Taxon	Representative Genera	Metabolite	Immunomodulatory Effects	References
Firmicutes	*Clostridium*	Acetate, Propionate, Butyrate, Tryptamine, Indole	Acetate: Inhibits the growth of pathogens, regulates cecal T cells and suppresses inflammasome activation. Propionate: Enhances intestinal epithelial barrier function, exerts anti-inflammatory and antioxidant effects. Butyrate: Induces the production of AvBDs, alleviates intestinal inflammation; inhibits HDACs and modulates the immune system. Tryptamine: Inhibits defensin expression (remains unconfirmed in poultry), strengthens barrier function, and modulates the immune system. Indole: Promotes defensin expression (remains unconfirmed in poultry), maintains intestinal homeostasis, relieves inflammation and improves mucosal barrier function. Vitamins: Modulate the immune system and exert anticoccidial effects. Lactic acid: Inhibits the proliferation of harmful bacteria, enhances intestinal barrier function, and modulates the secretion of antibodies and cytokines.	[40,41,42,43]
	*Lactobacillus*	Acetate, Propionate, Butyrate, Vitamins (B-complex vitamins, etc.), Indole, Lactic acid		[42,44,45,46,47]
	*Ruminococcus*	Tryptamine		[43]
	*Eubacterium*	Acetate, Butyrate		[48]
	*F* *a* *ecalibacterium*	Acetate, Propionate, Butyrate		[49]
Bacteroidetes	*Bacteroides*	Acetate, Propionate, Vitamins (vitamin K, B-complex vitamins, etc.), Indole		[42,50,51]
	*Parabacteroides*	Acetate, Propionate, Butyrate, Indole		[52,53]
	*Prevotella*	Acetate, Propionate, Indole		[54]
Actinobacteria	*Bifidobacterium*	Acetate, Vitamins (B-complex vitamins, etc.), Indole, Lactic acid		[42,55,56,57]
Proteobacteria	*Escherichia*	Acetate, Propionate, Butyrate, Indole, Vitamins (B-complex vitamins, etc.)		[42,58,59]

* the gut metabolites mentioned in this table have been studied in non-chicken models.

**Table 2:** Microbial metabolites that regulate defensins.

Microbial Metabolites	Receptor/Pathway	Defensin Affected	Species/Model	Evidence Type	Validated in Poultry
Tryptophan	mTOR signaling pathway	BD-2	Rat	In vivo	No
Kynurenine	AhR	BD-2	Mouse	In vitro and in vivo	No
Kynurenine	CaSR-AMPK pathway	pBD1, pBD2	Pig	In vitro and in vivo	No
I3C	-	BD-1	Mouse	In vitro and in vivo	No
L-Arginine	TLR4/NF-κB, MAPK, mTOR pathway	Porcine intestinal β-defensins	Pig	In vitro and in vivo	No
BCAAs	Sirt1/ERK/90RSK, G protein-coupled receptor-ERK pathway	Porcine intestinal β-defensins, HBD-2	Pig and human	In vitro and in vivo	No
Butyrate	-	AvBDs	Chicken	In vitro and in vivo	Yes
Butyrate + FSK	-	AvBDs	Chicken	In vitro and in vivo	Yes
Butyrate + FSK/cAMP	MAPK (ERK/JNK/p38) pathway	AvBD9	Chicken	In vitro and in vivo	Yes
Butyrate + Dietary sugars	MAPK, NF-κB, cAMP	AvBD9	Chicken	In vitro	Yes
Acetate + Propionate + Butyrate	-	AvBD9	Chicken	In vivo	Yes
SCFAs	mTOR, STAT3 pathway	β-defensins	Mouse	In vitro	No
Butyrate	HDAC inhibition	Porcine endogenous HDPs	Pig	In vitro	No
Propionate, Butyrate	High-concentration inhibition	α-defensin 5	Human	In vitro	No
DCA + NaB	-	AvBD3, and AvBD9	Chicken	In vitro	Yes
DCA	TGR5/NF-κB pathway, FXR, and CAR	HBD-1, and HBD-2	Human and mouse	In vitro and in vivo	No
Quercetin	NF-κB/p38 MAPK/cAMP pathway	AvBD6, AvBD12, and AvBD9	Chicken	In vivo	No
Resveratrol	COX-2 pathway inhibition	AvBD9	Chicken	In vitro	No
Curcumin	Modulate the microbial composition	AvBDs	Chicken	In vivo	No
PGN	TLR2/NOD1 Receptor, NF-κB/p38 MAPK, JNK pathway	AvBD9	Chicken	In vitro	Yes
5-HT	5-HT7 Receptor, ERK1/2 pathway, and PPAR-γ inhibition	HBD-1, and HBD-2	Human	In vitro	No

## Data Availability

No new primary data were generated in this study. All evidence supporting this review is available from the cited literature and publicly accessible sources referenced in the manuscript.

## Abbreviations

The following abbreviations are used in this manuscript:

5-HT	5-hydroxytryptamine
AhR	Aryl hydrocarbon receptor
AIV	Avian influenza virus
AMPs	Antimicrobial peptides
AvBDs	Avian β-defensins
BCAAs	Branched-chain amino acids
BD	β-defensin
CaSR	Calcium-sensing receptor
DCA	Deoxycholic acid
FXR	Farnesoid X receptor
GIT	Gastrointestinal tract
GPRs	G-protein coupled receptors
HDACs	Histone deacetylases
LTA	Lipoteichoic acid
MAPK	Mitogen-activated protein kinase
mTOR	Mammalian target of rapamycin
SBAs	Secondary bile acids
SCFAs	Short-chain fatty acids
SE	*Salmonella* Enteritidis
TGR5	Takeda GPCR5
